# Serum leptin differs in children with obstructive sleep apnea: A meta-analysis and PRISMA compliant article

**DOI:** 10.1097/MD.0000000000030986

**Published:** 2022-10-14

**Authors:** Yao He, Liu-Qing Zhou, Yao Hu, Qing Cheng, Xun Niu

**Affiliations:** a Department of Otorhinolaryngology, Union Hospital, Tongji Medical College, Huazhong University of Science and Technology, Wuhan, China; b Department of Otorhinolaryngology, The Central Hospital of Wuhan, Wuhan, China.

**Keywords:** cardiovascular disease, children, leptin, meta-analysis, obstructive sleep apnea

## Abstract

Obstructive sleep apnea (OSA) as an independent cardiovascular risk factor has been proposed, but the mechanisms underlying cardiovascular disease is far from being completely elucidated. Leptin, an inflammatory cytokine produced by adipocytes, contributes to the modulation of metabolism, respiratory control, and inflammation, which are factors associated with cardiovascular disease. Serum levels of leptin in children with OSA have shown conflicting results in previous studies.

**Methods:**

We performed a meta-analysis to clarify the correlation between leptin expression of the OSA patients following the PRISMA. PubMed, Embase, and Web of Science were systematically searched for relevant studies, and then independently screened by two researchers, and analyzed the data through STATA version 12.0.

**Results:**

In a total of 5 articles including 469 participants, the data analysis showed that serum leptin levels were elevated in children with OSA (MD, 6.36; 95% CI, 0.24–12.49, *P* < .001), compared to the control group. Subgroup analysis were performed based on body mass index. The results of subgroup analysis demonstrated that the serum leptin concentration was correlated with body mass index in children with OSA (MD, 9.70; 95% CI, 0.22–11.18, *P* < .001).

**Conclusions:**

The serum leptin levels were elevated in children with OSA, compared to the control group. It could add to our developing understanding of the pathogenesis and potential treatments for children with OSA, and help us to recognize the relevance of OSA in determining cardiovascular issues among children.

## 1. Introduction

Obstructive sleep apnea (OSA) is a disease that affect patients from infancy to adulthood. It affects 1% to 6% of all children and almost up to 59% of obese children.^[1]^ Untreated OSA is associated with neurobehavioral problems, decreased attention, disturbed emotional regulation, decreased academic performance, nighttime enuresis, impaired growth and so on.^[2,3]^ At last, OSA has a relevant impact on the cardiovascular system in children.^[4]^ Hence, early diagnosis of OSA may reduce the occurrence of systemic complications. At the same time, it is also important to explore the mechanisms of causing cardiovascular disease in children with OSA.

Leptin is an adipocyte-derived hormone regulating energy expenditure and food intake and can be found in the circulation.^[5]^ leptin, particularly in the context of hyperleptinemia, exerts detrimental effects in cardiovascular function and promotes adverse outcomes in cardiovascular disorders.^[6]^ Several studies reported the association between OSA and leptin.^[7–9]^ Dalesio et al showed that leptin was significantly higher in the obese/OSA group than in the control or OSA-only group.10 The association between OSA and serum leptin levels is intricate and multidirectional since leptin levels can also be affected by obesity alone.^[10]^ It is reported that leptin may play roles in early diagnosis of OSA.^[11]^ Alteration in the levels of leptin is associated with the risk of cardiovascular diseases.^[12]^ However, contrary results was showed by Li et al that leptin levels were not different between OSA and non-OSA groups.^[13]^ The variability of serum leptin levels in children with OSA remains clearly inconclusive. In the present study, the databases of PubMed, Embase and Web of Science were searched for relevant publications and a meta-analysis was performed. We tried to clarify whether serum leptin level is elevated in children with OSA.

## 2. Methods

### 2.1. Search strategy

We searched for English articles included in PubMed, Embase, and Web of Science. Search terms included the following key words: (obstructive sleep apnea hypopnea syndrome or sleep apnea or obstructive sleep apnea or obstructive sleep hypopnea or sleep-disordered breathing) and (adipokines or leptin) and (child or preschool children or teenager or adolescent). Potentially relevant articles were evaluated for inclusion against pre-specified eligibility and exclusion criteria.

### 2.2. Selection criteria

Eligible studies for the meta-analysis were required to meet the following inclusion criteria: participants at an age of 18 years or younger; serum leptin concentration was measured from morning fasting venous blood following overnight polysomnography; subjects received monitoring by polysomnography, and OSA was diagnosed if they had an obstructive apnea index ≥1. The exclusion criteria were the following: studies without sufficient data for meta-analysis; abstracts, reviews, letters, and case reports.

### 2.3. Statistical analysis

Statistical analyses were performed by using STATA version 12.0. Weighted mean difference (WMD) and 95% confidence interval (CI) were used to present the statistical results for continuous outcomes. And an inverse variance method was used for continuous variables.^[14]^ The level of statistical significance was set at *P* < .05.

Statistical heterogeneity was assessed on the basis of I square (I^2^) value. *P* < .10 was considered statistical heterogeneity to be statistically significant. An I^2^ value above 75% indicated high heterogeneity, an I^2^ value between 50% and 75% moderate heterogeneity, and an I^2^ value between 25% and 50% low heterogeneity. A result was believed to be homogeneous when an I^2^ value was <25%.^[15]^ If I^2^ <50%, the study was believed to be homogeneous or to have low heterogeneity, and the fixed effects model was used to pool the results. If I^2^ >50%, the study was believed to be moderately or highly heterogeneous, and the random effect model was used to pool the data.^[16,17]^

Subgroup analysis was done to assess the impact of body mass index (BMI ≥25 vs <25), and apnea-hypopnea index (AHI ≥10 vs <10). Sensitivity analysis was used to evaluate the stability of the meta-analysis results. Potential publication bias was evaluated by using funnel plot,^[18]^ the Begg test,^[19]^ and the test of Egger.^[18]^

## 3. Results

### 3.1. Search results

#### 3.1.1. Characteristics of the eligible studies

**Figure 1.**
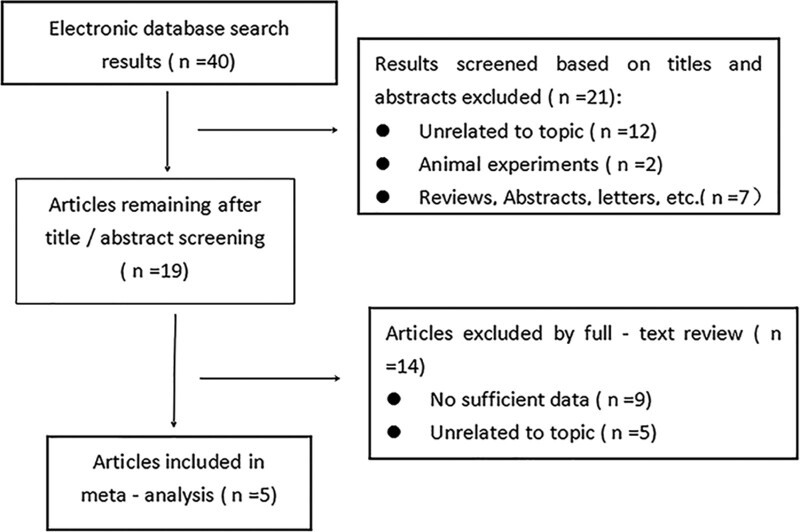
Flow diagram indicating the literature selection process and results included in the meta-analyses.

Overall 40 relevant articles were extracted from the databases. And 19 articles were identified to be relevant by roughly screened in terms of abstract and title against inclusion and exclusion criteria. We retrieved the full texts of the articles and excluded several full texts for following reasons: 5 had no control or control group was selected as AHI ≥5 events/hour in children; two had no basic information; 2 records included patients someone older than 18 years; 5 studies unrelated to topic. The steps of the literature search are detailed in Figure 1.

Finally, five studies covering data from a total of 469 participants, were included in this meta-analysis.^[10,13,20–22]^ The characteristics of the included studies were summarized, such as author, year, country, age, and so on. And the information of BMI, AHI and leptin of each study are given in Table 1. All value expressed as mean (± standard deviation).

**Table 1:** Characteristics of included studies and participants’ characteristics of included studies.

Study	Yr	Country	CG			0G					
			Leptin, ng/mL		n	Leptin, ng/mL		n	Age, yr	AHI, events/h	BMI, Kg/m^2^
			Mean	SD		Mean	SD				
Dalesio	2020	USA	7.4	8.8	18	28.8	21.3	13	7	≥10	≥28
Li (1)	2010	China	14.1	15.4	43	15.77	14.7	14	9.32	19.4	17.99
Li (2)	2010	China	48.38	23	55	41.27	20.7	29	11.66	11.2	28.02
Canapari	2011	USA	21.18	7.77	16	32.19	8.27	15	12.7	6.26	43.9
Bhatt	2021	India	9.5	3.5	57	19.2	8.2	190	10.71	13.3	27.1
Roche	2020	France, Brazil	65.91	31.78	8	54.14	20.37	11	15.4	6.2	39.6

AHI = apnea hypopnea index, BMI = body mass index, CG = control group, n = Sample size, OG = obstructive sleep apnea (OSA) group, SD = standard deviation.

#### 3.1.2. Pooled analysis

**Figure 2.**
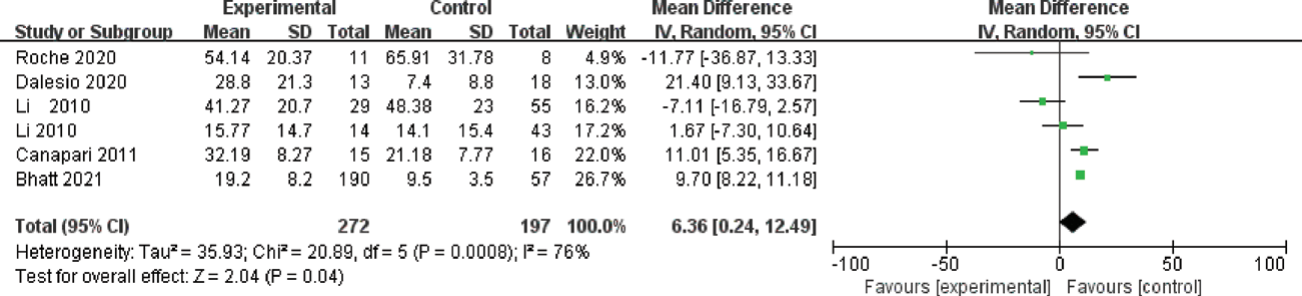
Forest plot and 95%CI for serum leptin levels in the OSA group in control with the control group in the meta-analysis. CI = confidence interval. OSA = obstructive sleep apnea.

The value of I^2^ was 76%, indicating that the studies were high heterogeneous. Therefore, the random effects model was used to combine effect size. Meta-analysis exhibited that serum leptin levels in OSA group were 6.36 ng/mL higher than that in control group (95%CI, 0.24–12.49, *P* = .04) Figure 2.

#### 3.1.3. Subgroup analysis - BMI

**Figure 3.**
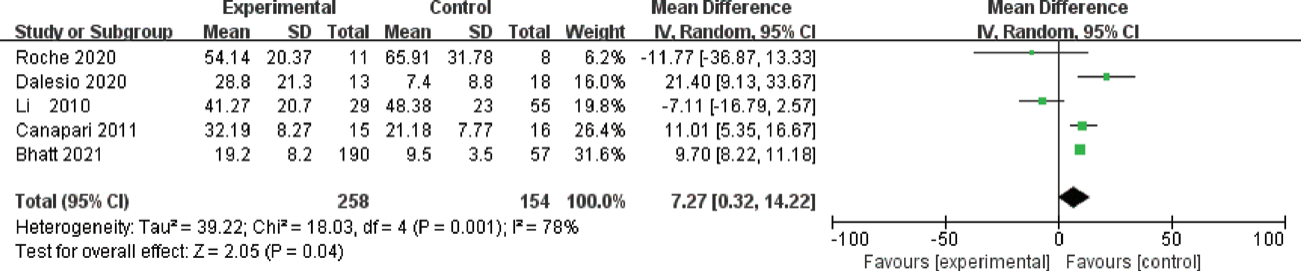
Subgroup analysis based on body mass index (BMI) >25.

All included studies reported the BMI values for each patient. Therefore, we performed a subgroup analysis of the articles according to obese or overweight. The total WMD in the studies with average BMI >25 was significant, with a corresponding value of 7.27 (95%CI, 0.32–13.22, *P* = .04; Fig. 3). The results revealed that the leptin level was elevated more significantly in the obese OSA patients. We also performed a special meta-analysis regarding the AHI (*P* > .05) of the patients with OSA. Our analysis showed that the leptin level was not significantly correlated with the AHI of the patients (MD, 6.15; 95%CI, −2.97–15.28, *P* = .19)

#### 3.1.4. Sensitivity analysis

**Figure 4.**
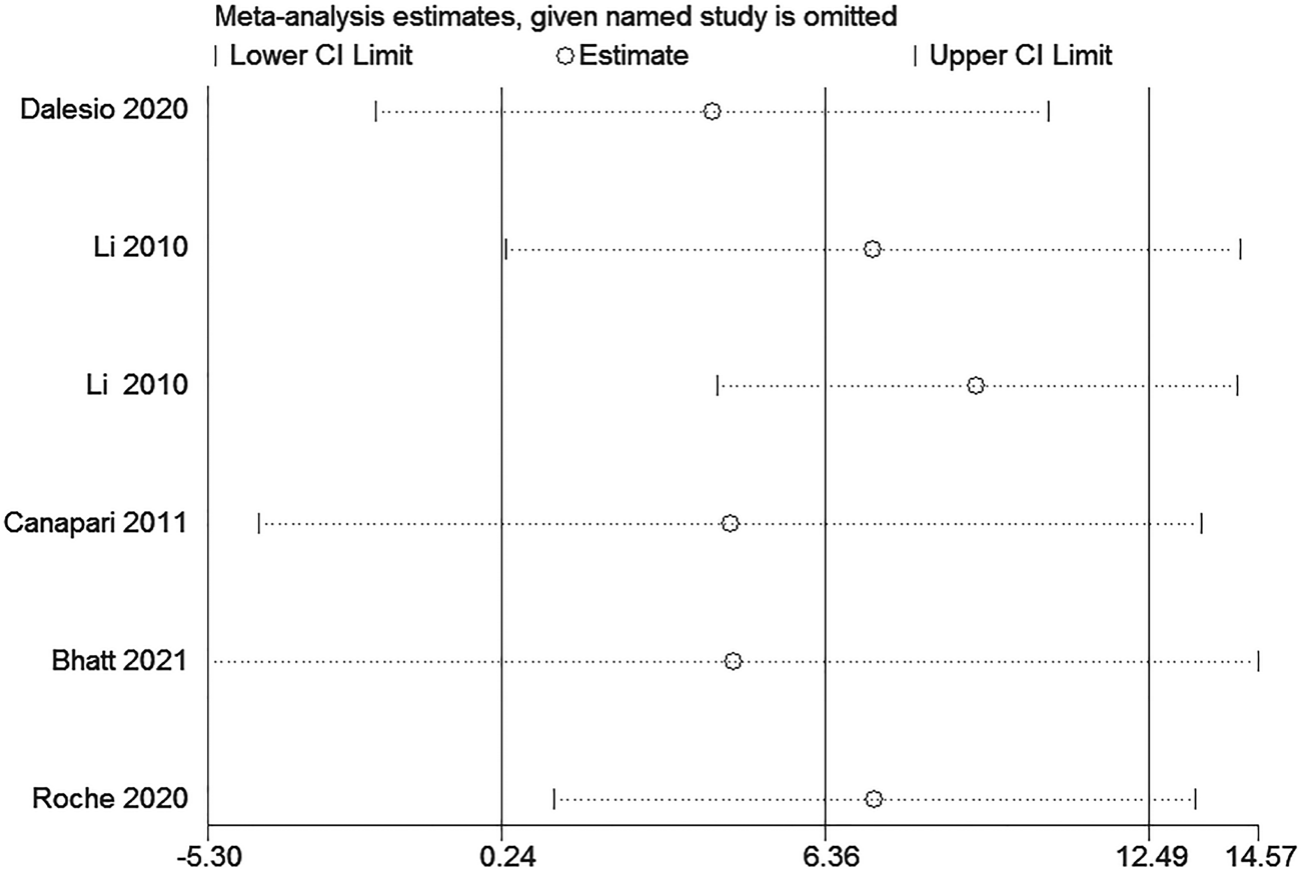
The sensitivity analyses were conducted to evaluate the effects of each single study on the overall effect.

We conducted a series of sensitivity analyses to explore the stability of the pooled data. The sensitivity analyses were conducted to evaluate the effects of each single study on the overall effect (Fig. 4). Based on the results of the sensitivity analysis, we again reviewed the inclusion literature, and ultimately, we remained these studies, the pooled effect size of the meta-analysis results was stable and reliable.

#### 3.1.5. Publication bias

We used Begg tests (*P* = .707) and Egger tests (*P* = .383) to assess publication bias in our study. The results were quite symmetric, indicating that the analysis did not include publication bias among the studies.

#### 3.1.6. Ethics

In this study, ethical approval was not necessary because the included data was based on previous published articles, and no original clinical data was collected or utilized.

## 4. Discussion

The present meta-analysis of 5 studies evaluated the serum leptin levels and compared these values between individuals with OSA and controls. The result showed that serum leptin levels were higher in children with OSA than in controls. And subgroup analysis revealed that the leptin level was significantly correlated with BMI (*P* < .001) which it was elevated more significantly in obese OSA patients. The overall heterogeneity was high in this meta-analysis (I^2^ = 76%); nevertheless, the sensitivity analysis indicated that no single study significantly influenced the pooled WMD, and Begg tests (*P* = .707) and Egger tests (*P* = .383) showed no publication bias in our study. Therefore, the result of our meta-analysis has relatively strong power. This is consistent with the previous findings that leptin levels appear to be determined by the degree of obesity as well as by the severity of OSA, particularly hypoxemia.^[8]^ The leptin is involved in energy expenditure, balance of blood glucose metabolism, inflammatory processes, and regulation of immune function. Animal studies demonstrated that leptin played key roles in respiratory control by acting centrally to alter ventilations.^[21]^ Moreover, leptin’s stimulation of oxidative stress, and at the cardiovascular level they result in vascular inflammation and vascular smooth muscle hypertrophy—all factors that contribute to atherosclerosis, hypertension, coronary heart disease and thrombosis.^[12]^ Sufficient clinical studies also showing that leptin levels are elevated in adult with OSA and associated with disease severity, age, and BMI.^[23–25]^ However, the consistency of the results regarding the leptin levels of leptin in children with OSA remains to be studied. Many studies have reported that patients with OSA have higher leptin levels than control groups.^[9–11,13,20,21,26,27]^ Both sleep deprivation and hypoxemia are thought to be critical causative factors in OSA-induced impact on leptin levels.^[28]^ Although, OSA and obesity are bidirectionally linked, One study demonstrated that serum leptin levels were significantly increases in children with OSA independently of obesity.^[9]^ Moreover, Treatment with continuous positive airway pressure (CPAP) and/or nasal corticosteroids in children with OSA led to a significant decrease in leptin while increases in leptin emerged in those with residual OSA.^[11,29]^ A more attractive hypothesis from these studies would be that changes in the frequency of intermittent hypoxia might have changed the serum leptin.^[30]^ This supports our conclusion from another dimension.

However, there are still some other sounds, such as leptin levels were not different between subjects with and without OSA.^[13]^ No associations between OSA severity and the blood level of leptin in this clinical sample of overweight and obese children and adolescents.^[31]^ These results differ from the results of this meta-analysis maybe because differences in the included populations and small sample size.

At last, the present meta-analysis contains several limitations. First, because of the differences in data statistics and experiment design, adequate data cannot be extracted from all or at least most of the included studies, numbers of articles were limited in the present meta-analysis. Second, obese children accounted for the majority of patients in the included studies which is associated with incidence of OSA in obese children. Third, the results may be heterogeneous due to the utilization of different methods and statistical analysis, sample size, and diagnostic work-up. Although, according to Begg’s funnel plots and the Egger’s test, the publication bias was not significant. So, according to present situation, additional studies with larger sample sizes are needed to acquire more representative and precise findings.

In conclusion, the meta-analysis demonstrated that serum leptin levels were elevated in children with OSA, compared to the control group. It could add to our developing understanding of the pathogenesis and potential treatments for children with OSA, and help us to recognize the relevance of OSA in determining cardiovascular issues among children.

## Author contributions

Qing Cheng, designed study, collected data, analyzed data, wrote article, revised article; Xun Niu, designed study, collected data, wrote article, analyzed data, revised article; Yao He, designed study, wrote article, analyzed data, revised article; Liu-Qing Zhou, designed study, analyzed data, revised article; Yao Hu, designed study, revised article.

**Data curation:** Yao Hu, Xun Niu.

**Formal analysis:** Xun Niu.

**Software:** Yao Hu, Qing Cheng.

**Writing – original draft:** Yao He.

**Writing – review & editing:** Liu-Qing Zhou, Qing Cheng.

## Correction

Qing Cheng’s name has been corrected from Qing Chen.
